# Lizards from Urban Areas Are More Asymmetric: Using Fluctuating Asymmetry to Evaluate Environmental Disturbance

**DOI:** 10.1371/journal.pone.0084190

**Published:** 2013-12-27

**Authors:** Marko M. Lazić, Antigoni Kaliontzopoulou, Miguel A. Carretero, Jelka Crnobrnja-Isailović

**Affiliations:** 1 Department of Biology and Ecology, Faculty of Sciences and Mathematics, University of Niš, Niš, Serbia; 2 CIBIO, Centro de Investigação em Biodiversidade e Recursos Genéticos, Universidade do Porto, Campus Agrário de Vairão, Vairão, Portugal; 3 Department of Ecology, Evolution, and Organismal Biology, Iowa State University, Ames, Iowa, United States of America; Monash University, Australia

## Abstract

The increase in human activities that leads to wildlife decline and species extinction poses an urgent need for simple indicators of environmental stress in animal populations. Several studies have suggested that fluctuating asymmetry (FA) can be an easy, direct measure of developmental instability because it is associated to environmental stress and, as such, it can be a useful indicator of population disturbance. We examined three different morphological traits in urban and rural populations of the common wall lizard (*Podarcis muralis*) to test whether anthropogenic disturbance causes an increase in FA. Compared to rural populations, urban ones showed higher levels of FA in all analyzed traits, thus providing evidence that FA can respond to anthropogenic disturbance. However, we also found significant differences in FA among traits, where femoral pores and subdigital lamellae, traits with a functional relevance, were more stable developmentally compared to supracilliar granules which have no evident function. Unsigned FA [abs(right-left)] exhibited significant, but weak, positive correlations among traits, indicating that developmental noise does not have a uniform effect across characters and thus questioning the view of developmental stability as an organism-wide property. The degree of signed FA (right-left) was more similar between structurally associated traits, possibly as an outcome of morphological integration. In conclusion, our results demonstrate that FA can be a reliable indicator of disturbance provided that it is analyzed on multiple traits simultaneously and examined at the population level.

## Introduction

Environmental stress (considered here as those environmental changes disrupting homeostasis and, ultimately, leading to decline in fitness of an individual) caused by human activities can have significant detrimental effects on animal populations [1], [2]. Anthropogenic pressures have been increasing in the past decades and they are affecting wildlife at all levels of biological organization, often leading to population decline and even to the extinction of entire species [3]. It is therefore crucial for conservation biologists to have a sensitive indicator, which can be implemented to detect signs of population disturbance before components of fitness have been affected and before irreversible demographic damage has occurred. In this context, a wide range of indicators of stress have been used in animals to detect disturbance by examining molecular, cellular, histological and/or morphological traits, at the individual or population level [4]. However, many of these are costly, time consuming and invasive. To enhance conservation practice, there is instead a need for efficient, easy to use, inexpensive and noninvasive indicators of population disturbance. Because developmental precision is affected by a wide range of environmental stressors, the degree of developmental instability (DI) has been suggested as a reliable indicator [5] that can be assessed using methodologies that fulfill the above requisites.

Fluctuating asymmetry (FA), defined as small, random deviations from perfect bilateral symmetry, has been widely accepted as a measure of developmental instability [6]. As the same genome controls the development of both the left and right side of bilaterally symmetrical traits and because both sides are developing in the same environment, increased levels of FA may indicate the inability of individuals to undergo precise development [7], [8]. Both environmental and genetic factors can influence developmental precision resulting in suboptimal phenotypes [9], [10] and influencing life history traits [11]. Due to this association, FA has been implemented as a sensitive indicator of stress levels in natural populations [5], [12].

Elevated levels of FA have been found both in laboratory experiments and in natural populations of plants and animals exposed to different types of stressors [13]–[15]. In many species pollution [16]–[18], extreme temperatures [19], [20], audiogenic stress [21], parasites [22]–[24], food deprivation [25], [26] and high population density [27] can disrupt developmental stability and increase FA. Additionally, inbreeding [28], [29], outbreeding [30] and hybridization [31] can also cause deviations from perfect symmetry. There are, however, studies where an association between FA and environmental or genetic factors could not be established [32], [33]. Further, many studies have shown that asymmetric individuals may exhibit lower fitness, demonstrating long-term effects of FA [34]–[36]. Yet, this is not always the case, as a lack of consequences on individual fitness associated to FA has also been reported [7], [32].

In lizards, high levels of FA have been associated with small island size and inbreeding [37], [38], habitat fragmentation [39], suboptimal incubation temperatures [40]–[43] and pollution ([44], but see [45]). Also, a correlation between FA and performance components potentially influencing fitness has been shown in *Psammodromus algirus* [46] and in *Iberolacerta cyreni* [47], where hindlimb asymmetry reduces running speeds. Moreover, association between FA and female choice was suggested to occur in *Iberolacerta cyreni*, where females were reported to prefer the scent of more symmetrical males [48]. Nevertheless, negative results have also been frequent. Island size had no effect on FA levels in *Podarcis muralis* [49], running speeds were similar between symmetric and asymmetric individuals of *Amphibolurus muricatus* [50], and female preference for symmetrical males was not detected in *Anolis carolinesis* [51].

Such contradictory results have led to question the validity of FA as a bio-indicator [52]. The association between fitness and FA can be nonexistent or weak, but it can also be the result of measurement error and small sample size; or it may be underestimated if asymmetries are analyzed considering only one trait [49], [53], [54]. Also, the strength of FA-fitness associations can also depend on stress levels. According to Clarke’s [55] early warning paradigm, fitness could decrease only under high stress and, as such, FA could serve as an early warning signal to infer population disturbance before fitness is actually affected [36]. Also, FA-fitness relations could be trait-specific, such that traits which directly influence fitness are expected to be more stable in their development and to remain unaffected even under high stress levels [56]. Additionally, under the developmental selection hypothesis [57], developmentally unstable individuals could perish under high stress levels before they reach adulthood, which would mask the FA-fitness association observed in adults. Finally, despite the conflicting results on FA-stress and FA-fitness associations, a recent meta-analysis [5], as well as general reviews on the subject [34], [55], suggests that overall FA is a valid indicator of stress-induced developmental instability and that it can be used in conservation biology as an early warning system.

**Figure 1:**
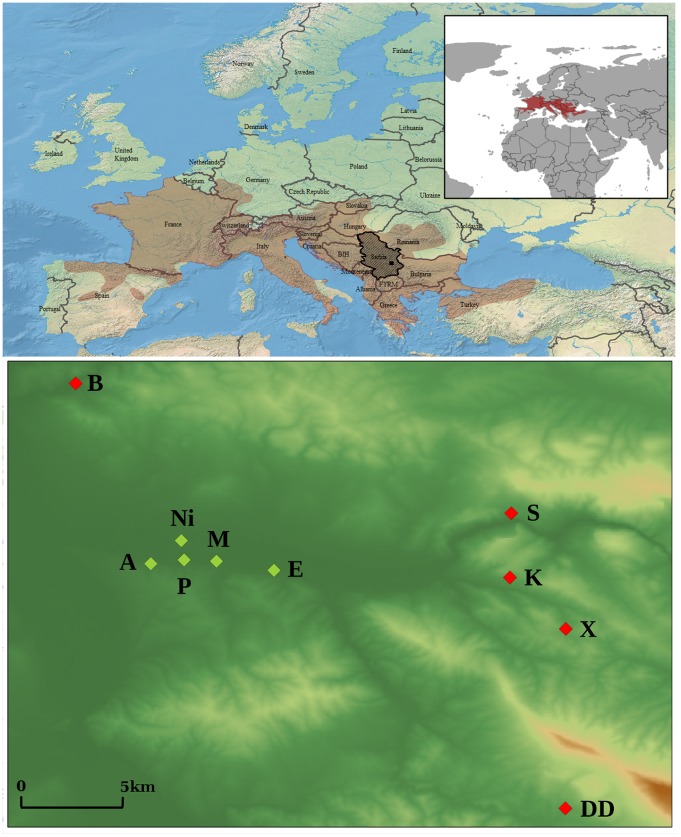
Distribution range of *Podarcis muralis* (top, shaded in brown; source: [59]), general location of the study area (top, black square) in Serbia (top, shaded in darker grey) and detailed distribution of sampling sites (bottom), where rural (green symbols) and urban (red symbols) populations were captured (altitude data source: [100]). See Table 1 for locality codes and sampling sizes.

In the present study we examined the degree of FA in three morphological traits (number of femoral pores, subdigital lamellae and supracilliar granules) in urban and rural populations of the common wall lizard, *Podarcis muralis* (Laurenti, 1768). This species has a great potential as a model organism for studying environmental disturbance in a broader context, as its populations are abundant and widespread across Europe (Figure 1; [58], [59]). We specifically hypothesize that, due to pollution and other disturbance factors, urban populations will show higher levels of developmental instability, as measured by FA, compared to rural ones. In order to explore this hypothesis, we followed a sequential design to address the following questions: 1) Do populations of the common wall lizard show FA in the examined traits, when taking measurement error into account? 2) Does FA vary across populations, sexes and traits? 3) If so, are these patterns consistent with an increased level of FA in urban populations? Additionally, we examined whether functional traits are more developmentally stable in which case they are predicted to show lower levels of FA compared to nonfunctional ones. Through this procedure we expect to quantify FA in a robust statistical framework and be able to establish whether it can be used for inferring local disturbance due to human activities in animal populations.

## Materials and Methods

### Study Sites

Adult individuals of *Podarcis muralis* were collected by noosing [60] between mid-April and July 2012. We sampled ten populations: five rural and five urban ones. The urban populations were all collected in different localities inside the City of Niš (southern Serbia), separated by straight distance between, 2 to 5 km approximately (Figure 1; Table 1), all at an altitude of about 200 m a.s.l. In these localities the lizards inhabited human-altered habitats and were directly exposed to urban contamination [61]. The five rural populations (Table 1) were all collected in the vicinities of Niš, at altitude range from 212 m to 487 m, and at a maximum distance of 30 km from the city center, in order to reduce the potential effects of geographic and genetic variation in our data. The broader area of Niš has a temperate continental climate, with average annual temperature of 11.2°C [62]. As for the populations collected in the city, lizards from rural populations generally inhabited human-constructed habitats (stone walls, houses etc.), as is usual for *P. muralis.* In contrast to the urban populations, however, these were localities with a very low level of air contamination and where human activities potentially creating pollution are few. The distribution of *P. muralis* can be considered continuous in the whole region with no obvious barriers neither between or within urban and rural populations (see below).

**Table 1:** Population type, collection localities, coordinates in datum WGS1984 and sample size (n) for males (M) and females (F) for all populations studied.

Population type	Population	Location	Coordinates	Sex	n
Urban	A	Kasarna “Bubanjski heroji”	43°18′39.87″N 21°52′48.39″E	M	23
				F	18
	E	“Electronic Industry – Niš”	43°18′26.21″N 21°57′2.05″E	M	12
				F	15
	M	Clinical Center Niš	43°18′44.93″N 21°55′3.63″E	M	18
				F	22
	Ni	Niš Fortress	43°19′27.39″N 21°53′51.23″E	M	22
				F	17
	P	Palilula ramp	43°18′47.57″N 21°53′56.98″E	M	22
				F	17
Rural	B	Paljine	43°24′51.25″N 21°50′13.75″E	M	25
				F	27
	DD	Donji Dušnik	43°10′15.74″N 22° 7′2.16″E	M	23
				F	17
	K	Kunovica	43°18′11.38″N 22° 5′7.94″E	M	20
				F	20
	S	Sićevo gorge	43°20′23.75″N 22° 5′10.62″E	M	18
				F	19
	X	Bancarevo	43°16′25.52″N 22° 7′3.00″E	M	20
				F	19

See also Figure 1.

### Trait Quantification

After capture, animals were transported to the laboratory of the Faculty of Sciences and Mathematics of the University of Niš, where they were sexed, weighed and measured. Snout-vent length was measured to the closest 0.01 mm using dial calipers. We considered as adults animals larger than 50 mm SVL [63] and with developed secondary sexual traits. According to Schulte [64], males were identified by the presence of hemipenises and by well-developed femoral pores on the internal side of the thighs. High resolution photos of functional traits, i.e. the femoral pores (FPN) and subdigital lamellae (SDLN) on 4^th^ toe of the hindlimb and the supracilliar granules (SCGN), which have no evident function, were taken on both sides of the body using a digital camera (Fuji Finepix S1600, resolution 12.2 MP). These traits were selected because they are easy and fast to quantify, and they present extensive variability in *Podarcis* populations and species [65]. For each individual, MML recorded the number of the aforementioned scale traits twice from the digital photos available, allowing several days of rest between the first and the second counting and randomizing the order of examined specimens, to ensure the independence of trait counts.

### Statistical Analyses

An asymmetry index (AI) was calculated for all examined traits as the value of the trait on the right side of the body, minus that on the left side (AI = R−L). AI values did not significantly deviate from normality within each population for any of the traits examined (Kolmogorov-Smirnov test, p>0.05 in all cases). Trait size dependence was examined by linear regression of unsigned AI values on SVL (to test for dependence on total body size) and on (R+L)/2 (to test for dependence on trait size). To test for the presence of directional and/or fluctuating asymmetry, while taking measurement error into account, we used a two-way ANOVA design on log-transformed trait values of each of the three traits separately, with side as a fixed factor, individual as a random factor and their interaction as an additional term. In this ANOVA design, a significant effect of “side” alone would indicate the presence of directional asymmetry (DA); a significant interaction between side and individual, would point to the existence of fluctuating asymmetry. ANOVA analyses were conducted separately for each population.

As our results indicated the existence of fluctuating asymmetry (FA) in all populations for all traits (see Results), we calculated an individual asymmetry index for each trait as the unsigned R−L difference between the log-transformed average of trait values across the two replicate counts of each individual, to account for measurement error (|ln(Raverage)−ln(Laverage)|) [66]. We then examined the effects of different factors on the degree of FA using an ANOVA design with sex, population type (urban vs. rural), population, nested within population type, and trait as factors and the individual FA index described above as the response variable. We also included all interaction effects.

We examined the correlation between traits in unsigned FA [abs(right-left)] to test whether asymmetry is organism-wide, in which case FA in a single trait can be used as an indicator of individual quality. We also examined the correlation observed in signed FA (right-left) to test whether traits developing in the same body part (e.g. FPN and SDLN, both located in the hindlimbs) show similar asymmetry patterns. This would be the case if perturbations are transmitted between associated traits during development [67].

All statistical analyses were conducted in STATISTICA version 8.0 [68].

### Ethics Statement

Lizards were collected and handled with permits of the Ministry of Environment and Spatial Planning of Republic of Serbia No.: 353-01-505/2012-03. All individuals were released in the capture sites upon completion of the procedures.

## Results

Linear regression of |R−L| on (R+L)/2 and SVL revealed no trait size or body size dependence for any of the analyzed traits (Table 2). Two-way ANOVAs applied to examine the effects of side and individual while taking measurement error into account, showed absence of DA in all populations for all traits. Measurement error was significantly lower than between-side variation (Table 3). AI values did not deviate significantly from normality (Kolmogorov-Smirnov test, p>0.05 in all cases) discarding antisymmetry.

**Table 2:** Statistical results obtained from linear regression of |R−L| on SVL and (R+L)/2 for all traits.

Trait	SVL			(R+L)/2		
	df	F	P	df	F	P
FPN |R−L|	1	0.738	0.390	1	0.017	0.869
SDLN |R−L|	1	0.179	0.666	1	0.714	0.389
SCGN |R−L|	1	1.233	0.172	1	1.160	0.185

Df: Degrees of freedom; F: F-statistic; P: corresponding P-value. See Material and Methods for variable abbreviations.

**Table 3:** Statistical results obtained from two-way, mixed model ANOVAs (side = fixed factor, individual = random factor) on log-transformed trait values, for all populations and traits separately.

			Individual effect			Side effect			Individual * side		
Population Type	Population	Trait	df	F	P	df	F	P	df	F	P
Urban	A	FPN	40	3.64	<0.0001	1	0.15	0.702	40	38.4	<0.0001
		SDLN	40	3.72	<0.0001	1	0.22	0.643	40	7.83	<0.0001
		SCGN	40	5.36	<0.0001	1	1.77	0.190	40	19.4	<0.0001
	E	FPN	25	6.06	<0.0001	1	1.50	0.232	25	173.64	<0.0001
		SDLN	25	3.10	0.003	1	0.80	0.382	25	25.5	<0.0001
		SCGN	25	3.34	0.0018	1	0.01	0.909	25	227.71	<0.0001
	M	FPN	39	3.53	<0.0001	1	0.74	0.394	39	70.17	<0.0001
		SDLN	39	4.67	<0.0001	1	1.53	0.222	39	8.15	<0.0001
		SCGN	39	3.03	<0.0001	1	0.53	0.471	39	42.87	<0.0001
	Ni	FPN	37	5.37	<0.0001	1	2.20	0.146	37	42.65	<0.0001
		SDLN	37	3.61	<0.0001	1	0.02	0.880	37	2.71	<0.0001
		SCGN	37	6.35	<0.0001	1	1.60	0.212	37	9.84	<0.0001
	P	FPN	39	2.44	0.0032	1	0.72	0.402	39	163.64	<0.0001
		SDLN	39	4.90	<0.0001	1	0.10	0.801	39	5.40	<0.0001
		SCGN	39	6.57	<0.0001	1	2.35	0.133	39	39.74	<0.0001
Rural	B	FPN	51	8.77	<0.0001	1	0.52	0.476	51	182.02	<0.0001
		SDLN	51	2.30	0.0014	1	2.10	0.153	51	26.90	<0.0001
		SCGN	51	4.82	<0.0001	1	0.59	0.445	51	28.89	<0.0001
	DD	FPN	39	7.94	<0.0001	1	1.10	0.300	39	127.38	<0.0001
		SDLN	39	5.96	<0.0001	1	1.58	0.215	39	4.73	<0.0001
		SCGN	39	3.67	<0.0001	1	2.31	0.136	39	19.27	<0.0001
	K	FPN	39	5.13	<0.0001	1	3.41	0.072	39	151.78	<0.0001
		SDLN	39	5.30	<0.0001	1	0.40	0.510	39	8.70	<0.0001
		SCGN	39	6.78	<0.0001	1	0.32	0.575	39	15.40	<0.0001
	S	FPN	36	6.72	<0.0001	1	0.26	0.615	36	151.76	<0.0001
		SDLN	36	4.30	<0.0001	1	0.00	0.867	36	8.30	<0.0001
		SCGN	36	7.72	<0.0001	1	1.69	0.201	36	41.97	<0.0001
	X	FPN	38	5.59	<0.0001	1	0.36	0.552	38	176.82	<0.0001
		SDLN	38	8.50	<0.0001	1	1.50	0.223	38	9.10	<0.0001
		SCGN	38	6.31	<0.0001	1	0.43	0.515	38	19.28	<0.0001

Df: Degrees of freedom; F: F-statistic; P: corresponding P-value. See Material and Methods for variable abbreviations, and Table 1 and Figure 1 for population codes.

**Figure 2:**
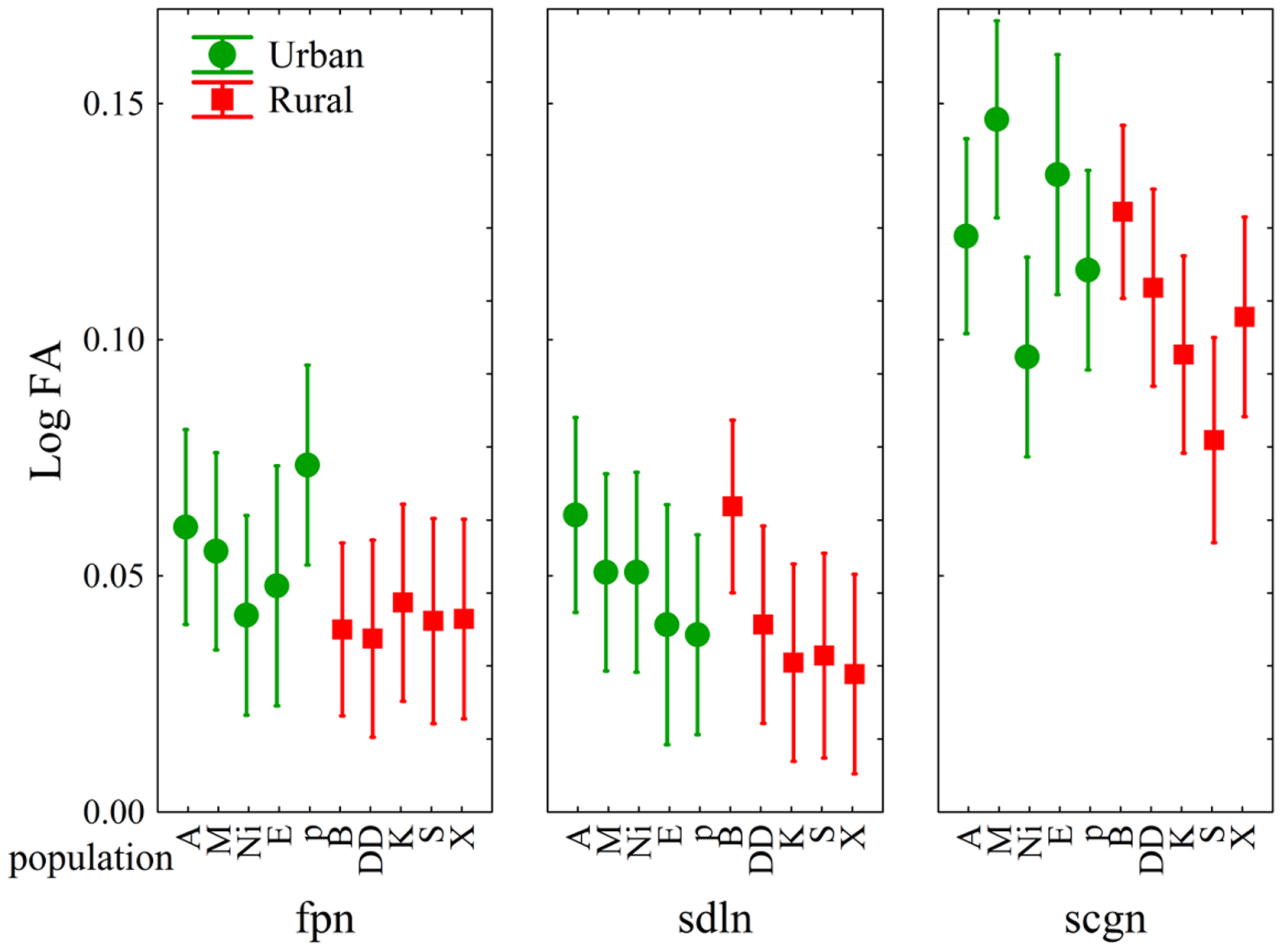
Mean degree of fluctuating asymmetry of femoral pores (FPN), subdigital lamellae (SDLN) and subciliar granules (SCGN) in urban (filled circles) and rural (open squares) populations of *Podarcis muralis* from southern Serbia. Error bars represent 95% confidence intervals. See Table 1 for locality codes and sampling sizes.

Three-way ANOVAs on the log-transformed average of trait values across the two replicate counts of each individual revealed differences both between populations and between population types (Table 4), where urban populations exhibited higher degrees of FA compared to rural populations. Differences across traits were also detected, where SCGN showed higher FA than both FPN and SDLN (Figure 2). There were no differences between the sexes in the level of FA and all interaction effects were also non-significant (Table 4).

**Table 4:** Statistical results obtained from three-way ANOVA on log-transformed average of trait values across the two replicate counts, with sex, population type (urban vs. rural), population nested within population type and trait as factors and all interaction effects.

	SS	df	F	P
Intercept	5.319353	1	1178.6	<0.0001
{1}PopType	0.056230	1	12.4	0.00043
Population(PopType)	0.084352	8	2.3	0.01723
{3}Sex	0.003984	1	0.8	0.347
{4}Trait	1.147046	2	127.0	<0.0001
PopType*Sex	0.007723	1	1.7	0.191
Population(PopType*Sex)	0.052748	8	1.4	0.167
PopType*Trait	0.004715	2	0.5	0.593
Population(PopType*Trait)	0.108073	16	1.4	0.093
Sex*Trait	0.016610	2	1.8	0.159
PopType*Sex*Trait	0.009119	2	1.0	0.364
Population(PopType*Sex)	0.052748	8	1.4	0.167
2(1*3*4)	0.046607	16	0.6	0.848
Error	5.063591	1122		

Significant, though weak, correlations were found between all traits in unsigned FA, and between FPN and SDLN in signed FA (Table 5).

**Table 5:** Pearson correlations between pairs of traits in signed and unsigned FA.

	Signed FA			Unsigned FA		
	FPNR−L	SDLNR−L	SCGNR−L	FPNR−L	SDLNR−L	SCGNR−L
FPN R−L		P = 0.026	P = 0.497		P<0.0001	P<0.0001
SDL R−L	r = 0.11		P = 0.631	r = 0.26		P = 0.0056
SCGN R−L	r = 0.03	r = −0.02		r = 0.18	r = 0.13	

Correlation coefficients are presented in below-diagonal elements and corresponding P-values in above-diagonal ones.

## Discussion

The results obtained throughout this study provide important insights for the study of asymmetries, both from a biological and a methodological perspective. As hypothesized, increased levels of FA were observed in urban populations of the common wall lizard compared to rural ones, supporting the idea that population-level FA can be used as an early indicator of environmental stress. However, our results also indicate that such inference requires the examination of FA in multiple traits at the population, not the individual, level, in order to obtain an accurate evaluation of developmental instability. This has important implications in the moment of using FA of morphological traits as a robust indicator of environmental disturbance for conservation purposes.

Both environmental and genetic factors can cause an increase in FA [8]. Heavy metals and other toxic chemicals can accumulate in adult females inhabiting polluted areas [69] and can be transferred to the eggs [70]. In species with permeable egg shells, pollutants can also be absorbed either from the soil in which the eggs develop or through gas and water exchange with the environment [71]. Sensitivity to environmental stressors, such as pollutants, is highest in the early phases of development and laboratory experiments have shown a significant effect of pollutants on embryos leading to abnormal development [72]. FA is thought to be a reliable indicator of disturbance, since many of the aforementioned pollutants are known to increase FA levels in various species. Unfortunately, there are few studies on lizards where effects of pollutants on developmental stability have been evaluated. High FA was found in populations of *Sceloporus occidentalis* inhabiting areas with high use of motor vehicles [44], but FA in femoral pores was found to be unaffected by a mixture of pesticides in *Podarcis bocagei* inhabiting agroenvironments [45].

High concentrations of heavy metals, especially lead and cadmium, have been found in the air [73], soil [74] and water [75] of the city of Niš. Contamination of air and soil with benzene, polycyclic aromatic hydrocarbons and persistent organic pollutants was also recorded [76] and comes from spilling and burning of 5000 m^3^ of oil and oil products from oil storage units (located less than four kilometers from the city center) and from fires in tobacco processing factories (less than two kilometers) in 1999, when high quantities of pollutants were released and spread across the city. Additionally, the increased use of motor vehicles and low quality of petrol also increase the emission of air pollutants (CO, HCOH, NOx and black smoke; [77]). Further, an important contributor to SO_2_ and NOx pollution is the district heating system with 13 heat source generators, which use crude-oil, heating oil, coal, and natural gas as fuels [78]. Under such environmental conditions developmental stability of embryos, either inside the mothers or inside the eggs laid, could be compromised as more energy is directed to physiological processes fighting pollution rather than to maintain developmental precision [11]. This is, therefore, a plausible explanation for the higher FA observed in populations of the common wall lizard in the city of Niš, which was consistent across different traits, independently of measurement error and variations among populations.

However, anthropogenic factors other than pollution may also have contributed to the increase of FA in urban populations. In ectotherms, temperature and water availability are major factors in development. Experiments with lizards have shown that, as incubation temperature increases beyond optimal levels, hatchling success decreases [79] and the level of FA increases [42]. It has also been shown that decreased water absorption during incubation reduces hatchling survival and fitness [80], but there are no data regarding its influence on developmental instability. Since temperatures are generally significantly higher [81] and humidity lower [82] in cities as compared to rural areas, temperature and water availability cannot be ruled out as factors contributing to high FA levels. Niš has a temperate continental climate, with maximum air temperatures in June, July, August and September exciding 35°C [83], [84].

Apart from pollution and temperature, loss of genetic variation due to inbreeding ([37], [38], [85]; but see [86]) and habitat fragmentation [39] has also been associated with increased developmental instability. Whether this is the case in urban populations of *Podarcis muralis* analyzed here is hard to know without data on genetic variability. However, lizards are widespread throughout the city and not restricted to small patches. Populations both within and around Niš are more or less connected, mainly through man-made structures such as railroads, as is frequently the case with *Podarcis muralis* [87], a fact that should ensure frequent gene flow. Further, although a decrease of genetic variation has been traditionally linked with anthropogenic pressures [88], [89], the strength and direction of this relation largely depend on the magnitude and type of stress [90]. While habitat fragmentation and high stress levels diminish, low stress levels have no effect and pollution even slightly increases genetic variation [90]. Additionally, Crnobrnja-Isailovic et al. [49] did not find a significant association between heterozygosity level and degree of FA in insular populations of *P. muralis*. Taking all this into account, the observed differences in FA levels between urban and rural populations of *Podarcis muralis* are more likely caused by physicochemical disturbance, rather than being the result of inbreeding via habitat fragmentation.

While this seems as the most feasible hypothesis, alternative explanations could be considered. Namely, with urban habitats being spatially less complex than natural landscapes, pregnant females, independently of pollution, could be forced to lay eggs in thermally suboptimal places. Also, predation pressure could be less intense (or simply different) in cities, allowing more asymmetrical individuals to survive to adult age or to get successful mattings [46], [48]. The second implicitly assumes adaptation, that is, that asymmetrical individuals could be negatively selected, due to their low condition, to asymmetry per se or to rejection by sexual partners. Both alternative hypotheses, even if unlikely, would require extra comparisons between juveniles and adults of all populations.

Although general FA patterns were concordant in all the traits examined, providing strong evidence for the existence of developmental instability in urban populations, the degree of FA visibly varied across traits. Specifically, SCGN exhibited higher FA values compared to FPN and SDLN across all populations. Consistent differences in FA between traits and across populations indicate differences in their developmental stability [67]. That is, developmental stability could be trait specific. The degree of FA may also be associated to trait functional significance, where traits with a high functional importance show lower FA [6], [91]. This is a plausible explanation for the patterns observed here. Femoral pores (FPN) are involved in reproductive signaling and acquisition of territory [92], [93], but also in intra- and interspecific recognition [94], [95]. Correspondingly, subdigital lamellae (SDLN) have been related to climbing capacity and are associated to habitat use [96], [97]. By contrast, supracilliar granules (SCGN) have no evident known functionality. Since both FPN and SDLN have important biological functions, their development could be under stricter control, as selection for developmental canalization is expected to be stronger in traits of functional significance [67].

Further, the developmental integration among traits might also explain variation in FA across different traits. Correlation analyses reinforce this view. Significant but weak correlations were found between all traits in unsigned FA, but only between FPN and SDLN when signed FA was examined. The significant correlation between FPN and SDLN in signed asymmetries suggests that there are interactions between these traits during development. This result comes as no surprise, as both traits are located in the same body part (hindlimbs) and perturbations during development can be transmitted between them [67]. In accordance with this hypothesis, association between levels of FA has been observed in the anterior and posterior wing regions of *Drosophila* flies suggesting strong developmental connection [98].

Further, correlations between trait asymmetries have been used to evaluate organism-wide developmental instability. If an individual asymmetry parameter (IAP) exists [91], [99], meaning that individuals that are asymmetrical for one trait tend to be similarly asymmetrical for other traits as well, then positive correlations in unsigned FA values of multiple traits are expected. If so, developmental instability could be seen as an organism-wide property and FA could be used as an indicator of individual quality. That has rarely been demonstrated, as trait FA correlations are usually low and non-significant [91]. In this study, correlations were significant, but the determination coefficients were very weak (explaining only 1.9%, 3.4% and 6.8% of variance, for FPN, SDLN and SCGN correspondingly). This suggests that, although some individuals tend to be systematically more asymmetric, background noise and extensive individual variation prevent us from using the asymmetry observed in a single trait and in individual lizards as a reliable indicator of disturbance. Instead, developmental instability should be evaluated using multiple traits and quantified at the population level.

Put together, our results provide evidence that FA responds to anthropogenic stress in populations of *P. muralis*. As such, it can serve as a sensitive bioindicator in this species. This conclusion is reinforced by the observation that general trends of FA, even if variable across populations, are consistently higher in urban localities as compared to rural ones, for several traits. However, the thorough cross-examination of different traits provides an important cautionary tale for conservation practice: methodological, developmental and/or evolutionary factors may cause a dissociation of FA levels across different traits and result in high levels of individual variation. As such, the examination of multiple traits at the population level should be a mandatory requisite, at least as a preliminary procedure, when using asymmetry patterns to establish environmental disturbance and implement conservation measures.
